# Understanding Health-Promoting Behaviors and Influential Factors in Schizophrenia Patients

**DOI:** 10.3390/nu16101490

**Published:** 2024-05-15

**Authors:** Heajin Yu

**Affiliations:** College of Nursing, Sahmyook University, Seoul 01795, Republic of Korea; heajinyu@syu.ac.kr; Tel.: +82-10-4494-1095

**Keywords:** schizophrenia, self-efficacy, health-promoting behaviors

## Abstract

(1) Background: People who are diagnosed with schizophrenia experience a reduced average lifespan compared to the general population. Also, approximately 85% of individuals with schizophrenia have chronic physical illnesses. Moreover, 60% of premature deaths in this population could be prevented through the adoption of health-promoting behaviors. (2) Methods: This study involved the recruitment of 220 participants from an outpatient clinic in Seoul, South Korea. Inclusion criteria comprised adults aged 19 years or older with a confirmed diagnosis of schizophrenia. Data collection occurred between 25 May 2021 and 2 August 2021, utilizing self-report questionnaires. A total of 202 responses were subjected to analysis using SPSS 23.0 and AMOS 23.0. (3) Results: The findings indicate that the final model is characterized by the following values: Normed *x*^2^ = 2.240, RMSEA = 0.079, TLI = 0.926, *x*^2^ = 562.2 (*p* < 0.001), AGFI = 0.830, GFI = 0.814, and CFI = 0.938. Notably, health knowledge did not exhibit a significant direct or overall impact on health-promoting behaviors. Conversely, social support and psychiatric symptoms demonstrated direct, indirect, and total effects on health promotion through an intervening variable. This study underscores the pivotal role of self-efficacy as the most influential factor affecting health-promoting behaviors in individuals with schizophrenia. (4) Conclusions: enhancing self-efficacy emerges as a crucial element in the design and implementation of intervention programs aimed at improving health-promoting behaviors in individuals with schizophrenia.

## 1. Introduction

Schizophrenia is a brain disorder in which the activation of dopamine is considered a major etiological factor. It can lead to symptoms such as hallucinations, delusions, disorganized speech, emotional blunting, and can also impair social functioning. The global lifetime prevalence of schizophrenia is approximately 1% (1 in 100 individuals), making it a relatively common mental disorder [1]. Individuals with schizophrenia have a lifespan that is on average 10 to 20 years shorter than the general population, and the premature mortality rate is about three times higher compared to age-matched peers [2]. Furthermore, 85% of individuals with chronic mental disorders, including schizophrenia, also have coexisting chronic physical illnesses. More than 60% of the causes of their premature mortality are related to preventable chronic physical illnesses [1]. Previous research indicates that prevalent chronic physical conditions such as cardiovascular diseases, hypertension, diabetes, and various respiratory conditions occur more frequently and at an earlier age in individuals with schizophrenia [3]. 

The primary reason for the higher prevalence of physical health problems in these individuals is their very poor lifestyle habits. Additionally, factors such as higher rates of smoking, alcohol consumption, physical inactivity, and inappropriate dietary habits, as well as the use of antipsychotic agents and genetic factors, contribute to their health issues [4]. Moreover, diagnostic overshadowing and other situations make it challenging to identify changes in physical symptoms. Therefore, it is crucial to not only focus on mental health but also pay attention to and manage the physical health of individuals with mental disorders, including schizophrenia, in order to improve their overall well-being and reduce premature mortality [5]. 

The relationship between mental and physical health is closely intertwined. Individuals with chronic mental disorders, as mentioned earlier, have a higher likelihood of being diagnosed with chronic physical illnesses [6]. Conversely, individuals with chronic physical illnesses experience mental health issues and emotional stress at a rate twice that of the general population. The coexistence of mental and physical conditions tends to decrease the quality of life and ultimately leads to health deterioration. Therefore, health management and promotion behaviors are crucial not only for the general population but also for individuals with mental disorders, especially those with schizophrenia [6].

The theoretical definition of health promotion behavior is ’individuals recognizing the importance of their health and engaging in preventive behaviors for health promotion.’ Health promotion behavior includes changing lifestyle habits or patterns through attitudes, knowledge, and behaviors related to health, thereby enhancing health management abilities [7]. Walker and Pender (1987) classified health-promoting behaviors into six categories: ‘health responsibility,’ ‘nutrition,’ ‘exercise,’ ‘stress management,’ ‘interpersonal relationships and self-realization.’ The Information–Motivation–Behavioral Skills Model (IMB), developed by Fisher and Fisher (1992) [8], is a psychological model that comprehensively explains health management behavior. It covers various health-related topics, including AIDS prevention, increasing physical activity for metabolic syndrome prevention, blood sugar management for diabetes patients, and health promotion behaviors among university students.

There is a significant need for empirical data to evaluate the health-promoting behaviors of individuals with schizophrenia, particularly within the context of a theoretical framework that thoroughly assesses these behaviors and their related factors. To address this need, this study utilizes a structural equation modeling (SEM) approach. SEM is a statistical technique that combines confirmatory factor analysis (CFA) and path analysis to empirically test a theory using collected data [9]. 

Among the various theories used to predict and explain health behaviors, such as the theory of planned behavior and the health-promoting model, the Information–Motivation–Behavioral Skills (IMB) model offers a comprehensive conceptualization of influencing factors for initiating and sustaining actions toward healthy behavioral changes. Key components of this model include acquiring sufficient information or knowledge about the situation, possessing motivation (both personal and social) for making behavioral changes, and possessing the necessary skills, including self-efficacy, to implement behavioral changes [8,10]. However, the application of this specific theoretical model to understand health-promoting behaviors and the factors influencing health promotion in individuals with schizophrenia remains an area requiring further exploration.

### Purpose

The purpose of this study was to validate the IMB model concerning health-promoting behaviors among individuals diagnosed with schizophrenia using a structural equation modeling (SEM) approach. The specific objectives were to (1) evaluate the extent of health-promoting behaviors in individuals with schizophrenia and (2) evaluate both the direct and indirect factors influencing health-promoting behaviors in this population.

## 2. Participants and Methods

This study had a cross-sectional, descriptive, and correlational research design, utilizing a structural equation modeling (SEM) approach to elucidate and forecast factors associated with health-promoting behaviors among individuals diagnosed with schizophrenia. Institutional Review Board (IRB) approval was secured before initiating contact with participants. Research participants were recruited from an outpatient psychiatric center in a metropolitan hospital in Seoul, South Korea, employing a convenience sampling technique, with research recruitment information disseminated through posters and leaflets in the outpatient clinic. Data were collected through self-report questionnaires from 25 May 2021 to 2 August 2021. 

Inclusion criteria encompassed adults (aged 19 or older) with a diagnosis of schizophrenia, while exclusion criteria applied to individuals with known neuro-cognitive disorders or those experiencing difficulties in communication due to an acute psychotic condition, verified by electronic medical records and the Mini Mental State Exam (MMSE) with a score below 24. Wolf (2013) [11] suggests that a minimum sample size of 200 is necessary for SEM to achieve parameter estimates with small standard errors, deemed practically useful. In a prior study by Seo (2001) [12] on the health-promoting behaviors of the elderly, a dropout rate of approximately 10% was established. Consequently, this study aimed to enroll a total of 220 participants.

Out of 243 individuals approached, 23 were deemed ineligible due to MMSE scores below 24. The remaining 220 participants meeting inclusion criteria provided informed consent, but 18 cases were excluded from analysis (3 dropouts and 15 with biased responses). Ultimately, data from 202 participants were included in the final analysis.

To mitigate potential biases during data collection, the researcher endeavored to select questionnaires that were as straightforward as possible for understanding and answering. Additionally, study participants were afforded ample time to complete surveys. Consistency was maintained by having only one researcher conduct participant recruitment and data collection. Demographic information and study variables were collected through self-reported surveys, either completed independently by participants or read by the researcher when necessary. Patient diagnoses and medication details were sourced from electronic medical records (EMRs). Participants received a small gift valued at 10,000 Korean Won (KRW) as a token of appreciation for the time and effort invested in completing the survey. 

### 2.1. Measurements 

Demographics: Demographic data, including age, sex, socioeconomic status, educational level, healthcare accessibility, community mental health welfare center registration status, and lifestyle, were gathered through a self-administered survey. Information on primary and secondary diagnoses, as well as a list of medications taken by participants, was obtained from electronic medical records. The daily antipsychotic dosage consumed by participants was converted into chlorpromazine equivalent dosage. The use of chlorpromazine equivalence (CPZE) has been a longstanding practice for comparing the dose and efficacy of both first- and second-generation antipsychotic agents [13]. In this study, age and sex, along with daily antipsychotic dosage, were designated as control variables.

Health knowledge: A measurement tool for health knowledge was used, the instrument developed by Ha (2005) [14], based on the 2001 Seoul Citizens’ Health Consciousness and Behavior Survey and the 2001 National Health and Nutrition Survey conducted by the Korea Institute for Health and Social Affairs. This measurement tool, consisting of 10 items, has been widely used to assess health knowledge across various age groups. Scores range from a minimum of 0 to a maximum of 10, with higher scores indicating higher health-related knowledge. Some sample questions are, ‘If you are obese, you are at a higher risk of developing diabetes and high cholesterol.’ When under stress, immune function weakens, making it easier to succumb to illness. In this study, Cronbach’s alpha was 0.89.

Social support: To measure social support among individuals with schizophrenia, the Medical Outcomes Study Social Support Survey (MOS-SSS) consisting of 19 items [15] was used. The Korean version of this tool, translated by Lim (2002) [16], was used in this study. Sample items are ‘Someone who shows you love and affection’ and ‘Someone to take you to the doctor if you needed it.’ The internal consistency was 0.95 in this study.

Self-efficacy: The Self-Rated Abilities Health Practices (SRAHP), a tool developed by Becker et al. (1993) [17] to measure health-related self-efficacy, consists of 24 items. The Korean version of the items adapted by Lee, Hong, and Park (2018) [18] was used in this study. Some sample items include ‘get help from others when I need it’ and ‘use medications correctly.’ In this study, Cronbach’s alpha was 0.96. 

Psychiatric symptoms: The Behavior and Symptoms Identification Scale-32 [19], consisting of 32 items, was used to assess psychiatric symptoms in individuals with schizophrenia. The Korean version of the items was employed in this study, which was translated and revised by Bae, Hong, and Shin (2011) [20]. This tool is a self-report measure of major symptoms and functional difficulties related to psychiatric disorders. Sample questions are ‘adjusting to major life stresses’ and ‘suicidal feelings or behavior.’ Cronbach’s alpha in this research was 0.97.

Health promotion behaviors: For the measurement of health promotion behaviors, the Health Promotion Lifestyle Profiles (HPLP) developed by Walker, Sechrist, and Pender (1987) [7] was used. The version adapted by Seo and Hah (2004) [21] was used. HPLP comprises 52 items and assesses health promotion behaviors across six domains: sense of responsibility for health (9 items), exercise (8 items), nutrition (9 items), spiritual growth (9 items), interpersonal relations (9 items), and stress management (8 items). Sample items include ‘eat 3–5 servings of vegetables each day’ and ‘reach my target heart rate when exercising.’ Cronbach’s alpha was 0.97 this study.

### 2.2. Statistical Analyses

The statistical analyses in this study utilized SPSS 23.0 and AMOS 23.0. Descriptive statistics were employed to explore participant characteristics, and the reliability of the scales was assessed using Cronbach’s alpha. A significance level of 0.05 (two-tailed) was set for all statistical tests. Model validation utilized the maximum likelihood estimate, assuming multivariate normality. Confirmatory factor analysis was conducted to validate the reliability of both exogenous and endogenous latent variables. Model fit was assessed through various indices, including the absolute fit (*x*^2^, goodness-of-fit index [GFI], Root-Mean-Square Error of Approximation [RMSEA]), incremental fit (Tucker–Lewis Index [TLI], Comparative Fit Index [CFI]), and Parsimonious Fit Index (Adjusted Goodness-of-Fit Index [AGFI], normed *x*^2^). To examine the significance of indirect and total effects, the bootstrapping technique was applied. This study presented two model fits for comparison: one excluding variables related to schizophrenia (psychotic symptoms and daily antipsychotic dosage) and the other incorporating these schizophrenia-related variables.

## 3. Results

The baseline characteristics of the study participants (*n* = 202) are outlined in Table 1. The average age of the participants was 48.7, ranging from 20 to 73. Of the participants, 54% were males and 46% were females. Approximately 30.7% of participants held a college degree or higher, while 57.9% graduated from high school. The majority (73.8%) were unemployed. Regarding health behaviors, 37.1% were current smokers, 17.3% were current drinkers, and 43.5% engaged in regular exercise. Most participants (86.2%) had a monthly or bimonthly psychiatric outpatient clinic visit, and only 19.8% utilized mental health welfare services. The daily antipsychotic dosage, calculated for both first- and second-generation antipsychotics using the Antipsychotic Dose Conversion Calculator from https://psychopharmacopeia.com/antipsychotic_conversion.php, ranged from 100 mg to 657.75 mg. Among the participants, 13.37% were on first-generation antipsychotics, while 99.5% were on second-generation antipsychotics.

The mean score for health knowledge was 7.99 ± 2.02, ranging from 0 to 10. Social support had a mean score of 53.06 ± 29.37, with scores ranging from 0 to 100. Psychiatric symptoms had a mean score of 65.74 ± 29.46, ranging from 32 to 160. Self-efficacy was reported with a mean score of 87.1 ± 23.36, ranging from 24 to 120. Health-promoting behaviors had a mean score of 126.44 ± 35.48, ranging from 52 to 208. The subscale scores for HPLP-II, from highest to lowest average scores, were as follows: health responsibility (2.49 ± 0.70), interpersonal relations (2.48 ± 0.76), spiritual growth (2.46 ± 0.70), stress management (2.44 ± 0.76), physical activity (2.37 ± 0.73), and nutrition (2.34 ± 0.79). 

The assumption of multivariate analysis was a normal distribution, and normality was divided into univariate and multivariate [22]. To test the univariate normality setting, skewness and kurtosis were used. To support the assumption of normality, the absolute value of skewness must be 3 or less, and 7 or less for kurtosis [23]. In this study, ranges for both skewness (1.499 to 1.114) and kurtosis (−0.173 to 3.131) of the measured variables were within the range required for the univariate normality assumption. According to Bae (2017) [22], if all values are within the satisfactory range in the results of the univariate normality test, it is acceptable to consider that the multivariate normality is also satisfied. Since the data in this study were considered to be normally distributed, the maximum likelihood method was used to estimate the parameters.

The diagnosis of multicollinearity typically involves the use of diagnostic tools such as the variance inflation factor (VIF) and tolerance. Generally, VIF values exceeding 10 or tolerances falling below 0.10 are indicative of multicollinearity [24]. In the present study, VIF values ranged from 1.3 to 1.9, and tolerance values ranged from 0.742 to 0.526. Additionally, when the absolute value of the correlation coefficient between variables exceeds 0.8, it suggests multicollinearity [24]. However, in this study, no evidence of collinearity issues was observed, with correlation coefficients ranging from −0.326 to 0.333.

The following are the results of the analysis of the finalized model. The fitness of the model is as follows: Normed *x*^2^ = 2.240, RMSEA = 0.079, TLI = 0.926, *x*^2^ = 562.2 (*p* < 0.001), AGFI = 0.830, GFI = 0.814, and CFI = 0.938 (Table 2).

### 3.1. Analysis of the Finalized Model

The following are the results of the analysis of the finalized model (Table 3). The health knowledge of individuals diagnosed with schizophrenia (β = −0.216, *p* = 0.002), social support (β = 0.317, *p* < 0.001), and psychiatric symptoms (β = −0.283, *p* < 0.001) demonstrated significant associations with self-efficacy, contributing to an explanatory power of 26%. On the other hand, factors such as age (β = 0.015, *p* = 0.827), sex (β = 0.025, *p* = 0.704), and daily antipsychotic dosage (β = 0.125, *p* = 0.072) did not exhibit significant relationships with self-efficacy. In terms of health-promoting behaviors, social support (β = 0.379, *p* < 0.001), psychiatric symptoms (β = −0.106, *p* = 0.057), and self-efficacy (β = 0.415, *p* < 0.001) were found to be significantly linked, explaining 50% of the variance. However, variables like health knowledge (β = 0.059, *p* = 0.315), age (β = 0.039, *p* = 0.491), sex (β = 0.097, *p* = 0.071), and daily antipsychotic dosage (β = 0.020, *p* = 0.732) did not exhibit significant associations with health-promoting behaviors.

### 3.2. Analysis of the Effect of Finalized Model

**Figure 1:**
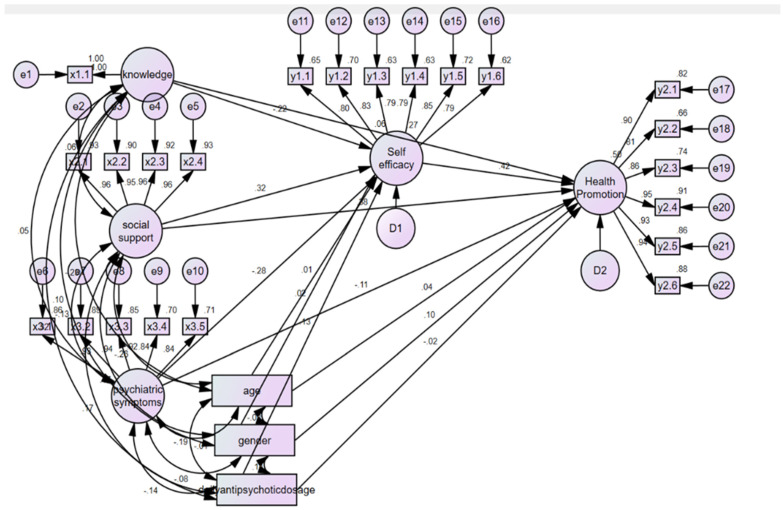
Path diagram of the finalized model.

The outcomes of the analysis, encompassing direct, indirect, and total effects of endogenous variables, are outlined in Table 4 and Figure 1. Health knowledge exhibited both a statistically significant direct effect (β = −0.216, *p* = 0.002) and total effect (β = −0.216, *p* = 0.002) on self-efficacy. Similarly, social support demonstrated significant direct (β = 0.317, *p* < 0.001) and total effects (β = 0.317, *p* < 0.001) on self-efficacy, while psychiatric symptoms exhibited significant direct (β = −0.283, *p* < 0.001) and total effects (β = −0.283, *p* < 0.001). Conversely, the direct and total effects of age (β = 0.015, *p* = 0.827), sex (β = 0.025, *p* = 0.704), and daily antipsychotic dosage (β = 0.125, *p* = 0.072) on self-efficacy were not statistically significant.

In terms of factors influencing health-promoting behaviors, social support demonstrated statistically significant direct (β = 0.379, *p* < 0.001), indirect (β = 0.132, *p* = 0.001), and total effects (β = 0.510, *p* = 0.001). The direct (β = 0.059, *p* = 0.315) and total effects (β = −0.031, *p* = 0.606) of health knowledge on health-promoting behaviors were not statistically significant. However, the indirect effect (β = −0.090, *p* = 0.005) of health knowledge on health-promoting behaviors was statistically significant. Psychiatric symptoms exhibited a statistically significant direct (β = −0.106, *p* = 0.057), indirect (β = −0.117, *p* = 0.001), and total effect (β = −0.224, *p* = 0.001) on health-promoting behaviors. The bootstrapping technique was employed to assess the significance of indirect effects, revealing that self-efficacy had a significant direct (β = 0.415, *p* < 0.001) and total effect (β = 0.415, *p* < 0.001) on health-promoting behaviors. None of the direct and total effects of age, sex, and daily antipsychotic dosage on health-promoting behaviors were found to be statistically significant.

### 3.3. Control Variables of Age, Sex, and Daily Antipsychotic Dosage

In multivariate analysis, age and sex are commonly adjusted for as confounding factors [25]. In this study, daily antipsychotic dosage was also included as a control variable. A control variable refers to any aspect held constant or restricted in a study that is not the focus of the research but is controlled due to its potential impact on study outcomes [22]. In this analysis, age, sex, and daily antipsychotic dosage were treated as exogenous variables, with single-headed arrows extending from the control variables to endogenous variables (dependent variables). Endogenous variables were connected to exogenous variables (independent variables) through double-headed arrows representing covariances in AMOS. This implies that age, sex, and daily antipsychotic dosage do not introduce confounding effects on the relationships with endogenous variables (self-efficacy and health-promoting behaviors). Prior to analysis, sex, being a categorical variable, was converted into a dummy variable. The findings indicated that all three control variables exhibited insignificant associations with endogenous variables (self-efficacy and health promotion), signifying that age, sex, and daily antipsychotic dosage do not introduce confounding effects on the relationships examined in this research (Figure 1).

## 4. Discussion

This research presents a comprehensive model that explains health-promoting behaviors in individuals with schizophrenia. As health promotion is essential for every individual, SEMs on health-promoting behaviors in the general population have greatly expanded. However, studies to date have not been designed to explain health-promoting behaviors for individuals with schizophrenia, although they are more vulnerable in managing their health than other populations. The present study contributes to the literature by taking the SEM approach to quantify the interrelated roles of assessing health-promoting behaviors and variables in individuals with schizophrenia by applying the IMB model. The finalized model demonstrates that, out of the seven total pathways, five of them were found to be statistically significant. Health knowledge did not have a direct or total effect on health promotion, yet it had an indirect effect. Social support and psychiatric symptoms both had direct, indirect, and total effects through the mediating variable (self-efficacy on health promotion). 

In explaining the health-promoting behaviors of individuals with schizophrenia, the research model using health knowledge, social support, and psychiatric symptoms as independent variables and self-efficacy as a mediating variable was acceptable when various model fit indices were considered. These results indicate the necessity for and importance of considering health knowledge, social support, and psychiatric symptoms, which have not been given much weight when examining the health promotion behaviors of individuals with schizophrenia. It is necessary to seek convergent and integrated interventions in the health and welfare areas when it comes to the approach based on the relationship structure between knowledge, social support, psychiatric symptoms, and self-efficacy when promoting the health of individuals with schizophrenia.

### 4.1. Health-Promoting Behaviors in Individuals with Schizophrenia

In this study, the mean score of health-promoting behaviors in individuals with schizophrenia was 126.44 ± 35.48 points, which was a moderate level (91–129). In comparison, the scores were higher in the general population without psychiatric disorders, for example, individuals with metabolic syndrome (141.96 ± 24.25) [26], male seafarers (136.14 ± 19.90) [27], and nursing students (137 ± 22.96) [28], and they were all in the good range (130–168). This supports previous findings indicating that individuals with SMIs, including schizophrenia, do not perform as well when it comes to health management, compared to the general population. They also live less healthy lifestyles compared to the general population [29,30]. These results suggest that developing intervention strategies to promote a healthy lifestyle is needed to encourage healthy behaviors in individuals with schizophrenia.

Among the six subscales of the HPLP-II, the lowest average score (2.34) was observed in the nutrition dimension in this study. This result is similar to research of Jo and Bang (2018), where the average score for nutrition was 2.9 in the general population (resort workers) and was lowest among all six subscales. Consuming non-nutritious food is a major and modifiable cause of CVD [31]. Individuals with schizophrenia have an especially difficult time consuming a healthy diet compared to the rest of the populace. In individuals with schizophrenia, adequate education on healthy eating habits is necessary, and healthcare professionals need to put effort into devising strategies to enhance the health-promoting behaviors of individuals with schizophrenia, especially for enhancing nutrition and maintaining a healthy diet.

### 4.2. Factors Related to Health-Promoting Behaviors in Schizophrenia

In this study, health knowledge was not associated with health-promoting behaviors independently, except through the influence of health knowledge on self-efficacy. In the study by [32], knowledge had an indirect effect on health promotion in male adults with T2D. Also, in the SEM research of [33], self-efficacy had a significant indirect effect on the relationship of health knowledge and prevention behaviors with dengue. It is suggested that increasing the health knowledge of individuals with schizophrenia is an essential first step toward promoting health. However, improved health knowledge alone is not enough, unless it results in an improvement in an individual’s level of self-confidence and self-efficacy.

Although it is difficult to say how knowledgeable one must be about health to achieve the desired health promotion, the impact of knowledge on health promotion has been validated across numerous studies [8,10]. Even though one may have adequate knowledge about health management, simply providing knowledge about health itself is not enough to drive changes in behavior. For example, most people know that chronic illness can be prevented by eating healthy foods and exercising regularly. However, only about 10% of adults in America eat the recommended amounts of fruits and vegetables, and only one out of every five U.S. adults exercise on a regular basis [34]. Tailored health education can enhance awareness of health and self-efficacy, which play major roles in increasing changes in behavior, including health management [35].

As observed in this study, the direct and total effect of health knowledge on health-promoting behaviors was not significant. This is comparable to the results seen in the literature, meaning that adequate health knowledge, when it comes to health-promoting behaviors, did not have a direct effect on the behaviors themselves, such as improving the participants’ eating behaviors or increasing their physical activity [36]. ’Health promotion’ is defined as a range of behaviors rather than a single behavior (e.g., an increase in physical activity, eating a healthy diet, and stress management). The consideration of multidimensional health-promoting intervention strategies is needed. It is believed that there is a limit to the amount of direct improvement to health-promoting behaviors that can be achieved solely through bestowing information regarding health management to individuals with SMIs.

Generally, one’s level of health knowledge influences self-efficacy and self-confidence [36,37]. In the study by [37], the level of knowledge regarding health-promoting behaviors had a positive effect on self-efficacy in correctional employees. However, the health knowledge of individuals with schizophrenia negatively affected self-efficacy in this study. This result is similar to a previous correlational study where it was suggested that negative feedback tends to lead to decreased self-efficacy, which is negatively associated with cognitive learning [38]. Individuals suffering from mental illnesses frequently face negative feedback in the form of stigma, avoidance, discrimination, false assumptions, and negative connotations, all of which can have a negative impact on their self-confidence/self-efficacy [39]. According to the findings, more research is needed to find ways to mitigate negative feedback toward people suffering from mental illnesses.

In this research, social support is shown to have direct, indirect, and total effects on health-promoting behaviors. This finding is similar to the results from a previous research study [40]. Favorable social support positively influenced the promotion and management of healthy behaviors in individuals with SMIs. Additionally, this finding replicated the results of a prior study that indicated obtaining adequate social support from family and friends as well as informational support from the community positively influenced health management in community-residing adults with schizophrenia [41].

In this study, the results show that the study participants had poor social support, with an average social support index score of only 53 out of 100. By comparison, adults living in rural South Korea had an average overall index score of 78.4 out of 100 in the social support study [42]. Schizophrenia patients often experience a lack of social support. These individuals may lack support from family, friends, or social networks, which can result from difficulties in social communication due to the illness, social withdrawal, or a lack of emotional support. However, it is important to note that this study was conducted during the pandemic, which may have exacerbated the lack of social support even further. Strengthening the social support of individuals with schizophrenia will provide a positive influence on health-promoting behaviors. This idea supports the previous study of Oh (2018) [43] that social support had a positive relation to health-promoting behaviors in older women living alone. Mental health services may consider that support groups for individuals with schizophrenia can foster a sense of connection and community for individuals with schizophrenia. As a result of this partial mediating effect, it can be seen that the social support of individuals with schizophrenia directly affects health-promoting behaviors while, at the same time, affecting health-promoting behaviors through the promotion of self-efficacy. If the mediating effect was found to be a complete mediating effect, regardless of how high the social support, it can be seen that health promotion activities are implemented only when self-efficacy is high. However, even if social support is high, this study can be interpreted as partially mediated by self-efficacy and affecting the implementation of health-promoting activities. Therefore, it can be interpreted that the higher the degree of self-efficacy of individuals with schizophrenia, the greater the possibility of affecting health-promoting behaviors.

Along with social support, psychiatric symptoms had direct, indirect, and total effects on health-promoting behaviors. The findings are similar to those in the literature. Adequate health knowledge, motivation, and social support tend to lessen psychiatric-related symptoms in adults with SMIs who lack effective symptom management [44]. Also, as previous studies show, well-managed psychiatric symptoms positively affect health management in individuals with SMIs [45,46]. Psychological intervention has shown that psychiatric symptoms were reduced in individuals with SMIs, including schizophrenia [47,48,49,50]. Along with promoting health, the consideration of utilizing psychological intervention needs to be provided. CBT seems to be effective in reducing psychiatric symptoms, especially patients with mild to moderate schizophrenia, when reduced psychiatric symptoms positively affect health-promoting behaviors [51].

Self-efficacy was found to be the strongest determinant of health-promoting behaviors in individuals with schizophrenia in this study. This result is similar to prior research studies that showed factors influencing health-promoting behavior. In the study of Stuifbergen and Becker (1994) [52], they confirmed that perceived self-efficacy was one of the strongest factors that positively influenced health management among adults with chronic, disabling conditions. Moreover, McAuley, Gothe, and Olson (2011) [53] stated that perceived self-efficacy is an essential factor that determines one’s willingness to engage in regular physical exercise. These results support the idea that self-efficacy can be improved through well-designed lifestyle modifications or health management programs, which in turn lead to promoting health.

In this study, self-efficacy demonstrated a partial mediating effect between social support and health-promoting behaviors in individuals with schizophrenia. This means that when social support influences health promoting behaviors, some of its effects occur through self-efficacy. Self-efficacy means that one continually attempts difficult tasks and always maintains a positive belief in one’s individual abilities [53]. This includes health-promoting behaviors. Individuals with a high level of self-efficacy tend to have high levels of health-promoting behaviors. This result is consistent with some previous literature which explained that individuals with mental illness who showed higher self-efficacy had better outcomes regarding health-promoting behaviors [54]. Furthermore, when one has a high level of social support, it leads to a high level of self-efficacy, which ultimately leads to a high level of health-promoting behaviors [55]. It is estimated that strong social support increases health-promoting behaviors. In other words, considering that self-efficacy is a positive belief in an individual’s ability, it is estimated that through the positive emotions created through adequate social support, individuals with schizophrenia will have positive feelings regarding their own confidence or challenging spirit, which will lead to more health-promoting behaviors.

This research study presents several limitations. First, this was a cross-sectional study that used self-reported measures, and cross-sectional studies are not able to infer causality among the measured variables. More research using longitudinal data is warranted. Second, the data were collected from a single psychiatric outpatient unit, which can mean that generalization of the samples may not be fully assured, when multisite approaches are needed. Third, even though the IMB model is considered to comprehensively explain health-promoting behaviors, other related factors, such as health beliefs and behavior intentions, were not considered. Fourth, the data were collected through a self-reported survey, which may lead to bias, such as recall bias and/or self-reporting bias.

## 5. Conclusions and Suggestions for Future Research

This study was conducted to construct and test hypotheses that apply the IMB model to individuals with schizophrenia. The specific aims were to measure health-promoting behaviors in individuals with schizophrenia and to identify the direct and indirect factors that influence health-promoting behaviors in individuals with schizophrenia. A total of 220 individuals with schizophrenia who were visiting the outpatient clinic in Seoul, South Korea, were considered, and the final data of 202 subjects were analyzed. The overall average score was 126.44 out of 208, and the score in the nutrition category was lowest among the six subscales. The finalized model explains that out of the seven total pathways, five pathways were found to be statistically significant. Health knowledge had an indirect effect on health-promoting behaviors. Social support and psychiatric symptoms had direct, indirect, and total effects on health-promoting behaviors through a mediating variable (self-efficacy).

Based on these results, it is necessary to find ways to promote activities and participation in order to improve the health-promoting behaviors of individuals with schizophrenia. Among the factors influencing health-promoting behaviors, the level of social support was notably lower than that of the general population. Policy considerations, such as establishing a welfare infrastructure to boost the level of social support for SMIs, are needed. Additionally, since the majority of schizophrenic patients are vulnerable to physical chronic diseases, it is necessary to develop and provide customized chronic disease self-management programs that take into account the characteristics of individuals with schizophrenia. Furthermore, active efforts by healthcare professionals are needed to conduct follow-up studies on improving the lifestyle of mentally ill patients and determining how to reduce the healthcare obstacles experienced by individuals with schizophrenia.

### Suggestions for Future Research

(1) A multi-site approach is needed when conducting studies related to health-promoting behaviors of individuals with schizophrenia.
(2) Additional research studies are needed that include additional factors explaining health-promoting behaviors.
(3) The development and validation of various intervention studies on enhancing health-promoting behaviors, especially healthy diets and nutrition for schizophrenia, need to be conducted.
(4) Additional intervention programs that enhance self-efficacy need to be created and implemented.

## Figures and Tables

**Table 1:** Characteristics of participants.

Characteristics	Categories	*n*	%
Sex (gender)	Male	109	54
	Female	93	46
Age (years)	19–29	6	3
	30–39	28	13.9
	40–49	59	29.2
	50–59	86	42.6
	≥59	23	10.9
	Mean ± SD		48.75 ± 9.504
Marital status	Married	24	11.9
	Single	142	70.3
	Divorced or separated	32	15.8
	Others	4	2
Education level	Elementary school	6	3
	Middle school	17	8.4
	High school	117	57.9
	College or above	62	30.7
Employment status	Unemployed	149	73.8
	Employed	53	26.2
Smoking status	Current smoker	75	37.1
	Nonsmoker	127	62.9
Alcohol consumption	Current drinker	35	17.3
	Nondrinker	167	82.8
Regular exercise	Yes	88	43.5
	No	114	56.5
First-generation antipsychotics		27	13.37%
Second-generation antipsychotics		201	99.50%
Antipsychotic dosage, chlorpromazine equivalents (mg)			Mean ± SD 596 ± 513

**Table 2:** The fitness of the model.

	*X*^2^ (*p*)	Normed *x*^2^	GFI	AGFI	RMSEA	TLI	CFI
Criteria	*p* > 0.10	≤3	≥0.90	≥0.90	≤0.80	≥0.90	≥0.90
Finalized Model	562.2 (<0.001)	2.240	0.814	0.830	0.079	0.926	0.938

GFI: Goodness-of-Fit Index, AGFI: Adjusted Goodness-of-Fit Index, RMSEA: Root-Mean-Square Error of Approximation, TLI: Tucker–Lewis Index, CFI: Comparative Fit Index.

**Table 3:** Standardized coefficient estimates of finalized model.

Endogenous Variable	Exogenous Variable	Estimate	S.E.	Standardized Estimates	C.R. (*p*)	SMC
Self-efficacy	Health knowledge	−0.441	0.142	−0.216	−3.102 (0.002)	0.274
	Social support	0.235	0.053	0.317	4.478 (<0.001)	
	Psychiatric symptoms	−0.248	0.060	−0.283	−4.126 (<0.001)	
	Age	0.001	0.006	0.015	0.219 (0.827)	
	Sex	0.043	0.113	0.025	0.380 (0.704)	
	Daily antipsychotic dosage	0.000	0.000	0.125	1.798 (0.072)	
Health-promoting behaviors	Health knowledge	0.092	0.091	0.059	1.006 (0.315)	0.500
	Social support	0.216	0.035	0.379	6.125 (<0.001)	
	Psychiatric symptoms	−0.072	0.039	−0.106	−1.831 (0.057)	
	Self-efficacy	0.319	0.054	0.415	5.951 (<0.001)	
	Age	0.003	0.004	0.039	0.689 (0.491)	
	Sex	0.129	0.071	0.097	1.805 (0.071)	
	Daily antipsychotic dosage	0.000	0.000	−0.020	−0.342 (0.732)	

S.E.: standard error; C.R., critical ratio; SMC: squared multiple correlation.

**Table 4:** Effects of predictive variables on endogenous variables in the finalized model.

Endogenous Variable	Exogenous Variable	Direct Effects	*p*	Indirect Effects	*p*	Total Effects	*p*
Self-efficacy	Health knowledge	−0.441 (−0.216)	0.002			−0.441 (−0.216)	0.002
	Social support	0.235 (0.317)	<0.001			0.235 (0.317)	<0.001
	Psychiatric symptoms	−0.248 (−0.283)	<0.001			−0.248 (−0.283)	<0.001
	Age	0.001 (0.015)	0.827			0.001 (0.015)	0.827
	Sex	0.043 (0.025)	0.704			0.043 (0.025)	0.704
	Daily antipsychotic dosage	0.000 (0.125)	0.072			0.000 (0.125)	0.072
Health-promoting behaviors	Health knowledge	0.092 (0.059)	0.315	−0.141 (−0.090)	0.005	−0.049 (−0.031)	0.606
	Social support	0.216 (0.379)	<0.001	0.075 (0.132)	0.001	0.291 (0.510)	0.001
	Psychiatric symptoms	−0.072 (−0.106)	0.057	−0.079 (−0.117)	0.001	−0.151 (−0.224)	0.001
	Self-efficacy	0.319 (0.415)	<0.001			0.319 (0.415)	0.001
	Age	0.003 (0.039)	0.491	0.000 (0.006)	0.824	0.003 (0.045)	0.483
	Sex	0.129 (0.097)	0.071	0.014 (0.010)	0.739	0.142 (0.107)	0.088
	Daily antipsychotic dosage	0.000 (−0.020)	0.732	0.000 (0.052)	0.087	0.000 (0.032)	0.601

## Data Availability

The data presented in this study are available upon request from the corresponding author. The data are not publicly available, due to respondents’ privacy.
